# Winkelstabile Platte zur Behandlung der Insuffizienzfrakturen des Beckens („minimally invasive posterior locked compression plate“): Videobeitrag

**DOI:** 10.1007/s00113-021-01039-x

**Published:** 2021-07-08

**Authors:** Imke Schmerwitz, Philipp Jungebluth, Stephan Bartels, Thomas Hockertz

**Affiliations:** Klinik für orthopädische Chirurgie, Sporttraumatologie und Unfallchirurgie, Städtisches Klinikum Wolfenbüttel, Alter Weg 80, 38302 Wolfenbüttel, Deutschland

**Keywords:** Insuffizienzfrakturen, Osteoporotische Beckenfrakturen, Winkelstabile dorsale Beckenplatte, Fragility Fractures of the pelvis, Angular stable pelvic plate, Fragility fractures of the pelvis, Osteoporotic pelvis fractures, Insufficiency fractures

## Abstract

**Operationsziel:**

Belastungsstabile Versorgung des Beckenringes mit hoher Primärstabilität.

**Indikation:**

Instabilität und ausbleibende Mobilisierbarkeit bei osteoporotischen Beckenbrüchen.

**Kontraindikationen:**

Dekubitalgeschwüre, Infekte.

**Operationstechnik:**

Minimal-invasive winkelstabile Versorgung durch 4,5 LCP (locked compression plate, DePuy Synthes, Zuchwil, Schweiz) von dorsal.

**Weiterbehandlung:**

Sofortige Mobilisation mit Vollbelastung, Röntgenverlaufskontrolle.

**Evidenz:**

Die Nachuntersuchung eines Kollektivs von 34 Patienten zeigte keine Implantatlockerungen sowie eine vergleichsweise niedrige Strahlenexposition.

**Video online:**

Die Online-Version dieses Beitrags (10.1007/s00113-021-01039-x) enthält das Video zur hier beschriebenen Operationstechnik „Winkelstabile Platte zur Behandlung der Insuffizienzfrakturen des Beckens“.

## Abstract (en)

**Objective of surgery:**

High primary stability of the pelvic girdle with full weight bearing.

**Indications:**

Instability and immobility in patients with osteoporotic fractures of the pelvis.

**Contraindications:**

Decubitus ulcers, infections.

**Surgical technique:**

Minimally invasive posterior locked compression plate 4.5 LCP (locked compression plate, DePuy Synthes, Zuchwil, Switzerland).

**Follow-up:**

Immediate mobilization with full weight bearing, X‑ ray control.

**Evidence:**

The follow-up examination of a collective of 34 patients showed no implant loosening and a relatively low radiation exposure.

## Hintergrund

Die Häufigkeit der Insuffizienzfrakturen des Beckens, auch als „fragility fractures“ bezeichnet, hat in den letzten Jahrzehnten deutlich zugenommen [1]. Zur operativen Versorgung dieser Frakturen kommt häufig die iliosakrale Schraubenosteosynthese zum Einsatz [3, 4, 7, 12]. Auch die Versorgung durch Transsakralstäbe, spinopelvine Abstützung, Fixateur externe und kombinierte Verfahren wird beschrieben [2, 5, 8]. Implantatlockerungen und Dislokationen werden in der Literatur in bis zu 18 % beschrieben [4].

## Definition, Klassifikation, Operationsindikation

Die Indikationsstellung zur Operation ergibt sich in Zusammenschau aus dem klinischen und radiologischen Bild. Alle Patienten mit Nachweis einer Beckenringfraktur auf dem konventionellen Röntgenbild des Beckens erhalten ein Becken-CT (Computertomograpie) zur Frakturbeurteilung. Die Frakturklassifikation erfolgt nach Rommens und Hofmann [10]. Bei fehlender Mobilisierbarkeit der Patienten, starken Schmerzen und/oder hochgradig instabilen Frakturen wird die Operationsindikation gestellt [9]. Unabhängig von der Frakturklassifikation erfolgt bei den Patienten, bei denen keine Weichteilproblematik besteht und die vor der Verletzung noch mobil waren, die Versorgung durch eine minimal-invasive dorsale Beckenplatte (MIPLCP). Bei schon vor der Verletzung immobilen Patienten ist die Operationsindikation, aufgrund möglicher postoperativer Weichteilkomplikationen, kritisch zu hinterfragen. Therapeutisch sollte hier die Versorgung mittels iliosakraler Schraubenosteosynthese vorgezogen werden. Durch die Winkelstabilität der Osteosynthese wird eine hohe Primärstabilität erzielt. Das Verfahren wird minimal-invasiv durchgeführt. Im Gegensatz zu der am häufigsten eingesetzten iliosakralen Schraubenosteosynthese wurden Implantatlockerungen bis dato nicht beobachtet, bei vergleichbaren Eingriffs- und niedrigeren Durchleuchtungszeiten [11]. Das Verfahren kann als „Stand-alone“-Verfahren eingesetzt werden; eine zusätzliche ventrale Versorgung ist nur sehr selten notwendig [6].

## Fallbeschreibung

Wir stellen den Fall einer 86-jährigen Patientin vor, die am 24.12.2020 in ihrer Wohnung gestürzt war und aufgrund von zunehmenden immobilisierenden Schmerzen am 27.12.2020 stationär aufgenommen wurde. In der bei der Aufnahme durchgeführten Beckenübersicht zeigte sich eine acetabulumnahe vordere Beckenringfraktur links. In der CT wurde darüber hinaus auch eine Beteiligung des hinteren Beckenringes festgestellt. Die Frakturen wurden nach Rommens und Hofmann in FFP IVb klassifiziert. Wesentliche Vorerkrankungen bestanden nicht. Im Aufnahmelabor zeigte sich an pathologischen Werten ein leicht erhöhtes CRP mit 26,5 mg/dl (in der präoperativen Kontrolle rückläufig auf 2,7 mg/dl) bei ansonsten normalen Laborbefunden. Klinisch klagte die Patientin über Schmerzen in der linken Leiste und über dem Sakrum. Auch unter suffizienter Schmerztherapie ließ sich die Patientin lediglich mit dem Rollator für wenige Schritte mobilisieren. Da sich unter konservativer Therapie auch am 5. Tag keine Besserung einstellte, willigte die Patientin in das ihr vorgeschlagene operative Verfahren ein. Vor der Verletzung hat sich die Patientin noch allein zu Hause versorgt und war gut mobil. Die operative Versorgung erfolgte mittels minimal-invasiver, winkelstabiler, dorsaler Beckenplatte (MIPLCP). Dieses Verfahren kommt bei uns primär zur Versorgung von Fragility fractures des Beckens zum Einsatz. Als Kontraindikation sehen wir eine Bettlägerigkeit an, die bereits vor der Verletzung bestand, sowie Weichteilprobleme (Dekubitalgeschwüre) oder vorliegende Infekte. Als Alternativverfahren kommt in unserer Klinik die iliosakrale Schraubenosteosynthese zur Anwendung. Durch die Versorgung mittels MIPLCP wird eine hohe Primärstabilität erreicht, und die Patienten sind postoperativ sofort unter Vollbelastung mobilisierbar. Insbesondere bei dem betagten Patientenkollektiv halten wir es für essenziell, die Immobilitätsphase so gering wie möglich zu halten. Die Patientin konnte ab dem ersten postoperativen Tag mit Vollbelastung mobilisiert werden. Die Wunden heilten primär. Zur weiteren Rehabilitation wurde sie am 10. postoperativen Tag in die Anschlussheilbehandlung entlassen. Bei der routinemäßigen Wiedervorstellung nach 8 Wochen war sie bereits im Wesentlichen ohne Hilfsmittel mobil und nahezu schmerzfrei. Analgetika wurden nicht mehr benötigt. Die Schrittgeschwindigkeit auf 4 m („gait speed“) betrug 5,6 s. Die im weiteren Verlauf durchgeführte konventionelle Beckenübersicht zeigte eine weiterhin regelrechte Implantatlage bei zunehmender Kallusbildung. Die CT-Abschlusskontrolle nach 6 Monaten steht noch aus.

## Operationsablauf

**Figure 1:**
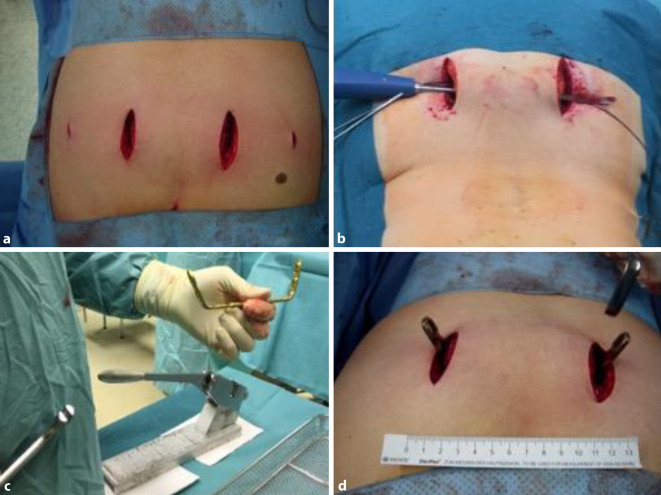

Der Erfolg des Verfahrens basiert zum einen auf der korrekt durchgeführten Osteosynthese, zum anderen auf einem sehr schonenden Umgang mit dem die Osteosynthese umgebenden Weichteilmantel. Die Operation wird in Bauchlage durchgeführt. Die Lagerung erfolgt auf einem röntgendurchlässigen Tisch, vorzugsweise auf einem Karbontisch. Vor dem sterilen Abwaschen und Abdecken des Operationsgebietes erfolgt eine Kontrolle der korrekten Lagerung und Röntgeneinstellung. Die erforderlichen Röntgeneinstellungen umfassen die Becken‑a.-p.-Ansicht, die Becken-Outlet-Aufnahme, um die Lage der Schrauben zu den Neuroforamina zu beurteilen, und die Inlet-Aufnahme, um ein ventrales Überstehen der Schrauben auszuschließen. Auch eine Zieleinstellung der IS-Fugen kann sich intraoperativ als hilfreich erweisen. Eine „Single-shot“-Antibiotikagabe als perioperative Prophylaxe ist obligat. Nach sterilem Abwaschen und Abdecken des Operationsgebietes werden bilateral 2 längs verlaufende Inzisionen von jeweils 4–6 cm Länge ungefähr 2 Querfinger lateral der Spina iliaca posterior angelegt. Nach Durchtrennen von Haut und Subkutangewebe wird die Faszie dargestellt. Die Faszie des M. glutaeus maximus wird scharf vom Os ilium abgesetzt und mit einem Haltefaden gefasst (1:10). Letzteres verhindert ein Abgleiten des Muskels nach ventral-lateral und ist im weiteren Verlauf hilfreich beim Verschluss des Situs. Der M. glutaeus kann sodann mit einem breiten Rasparatorium vom Ileum abgeschoben werden (1:45). Eine Beschädigung des Muskels sollte hierbei vermieden werden. Die freigelegte Fläche der Beckenschaufel sollte so groß sein, dass ein problemloses „Einschwenken“ der Platte möglich wird (s. unten). Nach medial wird nun die thorakolumbale Faszie von der Crista ebenfalls scharf gelöst (1:55). Mit einem langen Rasparatorium kann nun unterhalb der Faszie das Plattenlager geschlossen präpariert werden (2:05). Zur Stabilisation wird eine 4,5-mm-LCP-Standardplatte (DePuy Synthes, Zuchwil, Schweiz) verwendet. In der Regel kommt eine 11-Loch- oder 12-Loch-Platte zur Anwendung. Die Platte wird an beiden Enden im Bereich des dritten Plattenlochs auf beiden Seiten um ca. 55–60° zur Knochenseite gebogen (3:50). Die Platte wird dann mit ihrer konvexen Seite nach ventral durch den subfaszialen Tunnel geschoben (5:05). Nachdem die Platte die Gegenseite erreicht hat, kann sie um 180° gedreht werden (5:20), sodass sich die lateralen Enden dem dorsalen Aspekt des Os Iliums anlegen (Abb. 1a–d und 2a–c). Bei sehr kachektischen Patienten mit limitierten Weichteilverhältnissen kann die Crista iliaca auf die Breite und Dicke der Platte reseziert werden und die Platte so in der Crista „versenkt“ werden.

**Figure 2:**
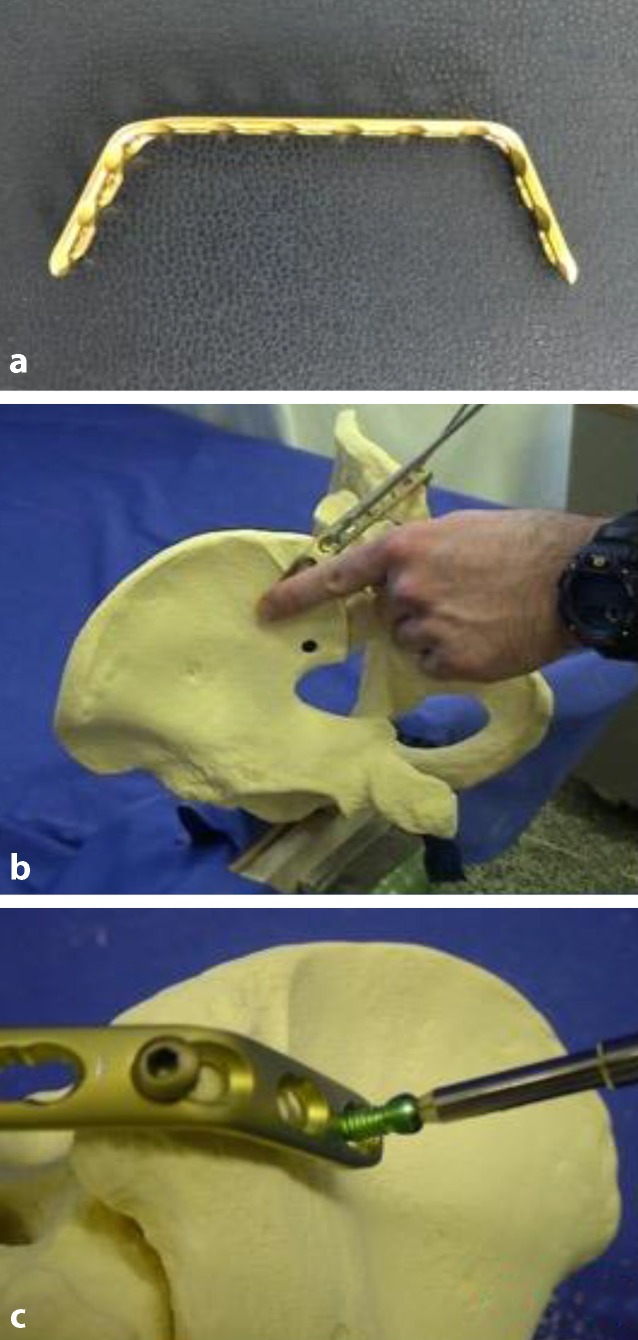

Jetzt erfolgt die radiologische Kontrolle der Plattenlage, ggf. kann eine Lagekorrektur vorgenommen werden. Die Platte liegt nun an den Biegungspunkten der dorsalen Crista iliaca an. Über die Plattenlöcher an der Biegungsstelle wird nun die Crista mit dem Bohrer eröffnet (5:50). Es werden Kortikalisschrauben von 65–80 mm Länge im Ilium platziert, mit deren Hilfe die Platte an den dorsalen Beckenring herangepresst wird. Sollte dieses Manöver bei Vorliegen einer extremen Osteoporose misslingen, muss die Platte manuell durch den Assistenten mithilfe eines Kugelpfriems platziert und dort gehalten werden. Die lateralen Plattenlöcher können nun mit winkelstabilen Kopfverriegelungsschrauben besetzt werden. Um eine sichere Verankerung der Schrauben in der Massa lateralis des Sakrums zu gewährleisten, müssen 3 Kortikalisschichten (2-mal Ilium, einmal Sakrum) passiert werden (7:30). Nach Durchbohren der ersten Kortikalis wird der Bohrer linksdrehend bis zur nächsten Kortikalis tastend vorgetrieben, welche dann wieder rechtsdrehend durchbohrt wird. Dieser Vorgang wird insgesamt 3‑mal wiederholt. Ein Durchbohren der vierten Kortikalis ist unbedingt zu vermeiden, da dahinter entweder das Neuroforamen oder ventral die präsakralen Strukturen verlaufen. Es finden Schrauben der Länge 36–50 mm Verwendung. Sollte beim Bohrvorgang für diese Schrauben der Bohrer mit der zuvor eingebrachten Kortikalisschraube kollidieren, kann dieses Loch zunächst monokortikal mit einer kurzen Schraube besetzt werden. Die nur zur Plattenpositionierung eingebrachte Kortikalisschraube kann entfernt werden. Es sollte keine Dislokation der Platte auftreten. Das zweite laterale Plattenloch kann nun besetzt werden, danach kann die kurze Schraube gegen eine entsprechend längere Schraube ersetzt werden. Nachdem die winkelstabilen Schrauben bilateral fixiert worden sind, sollten die Kortikalisschrauben entfernt werden, da sich diese sonst im Verlauf lockern können.

**Figure 3:**
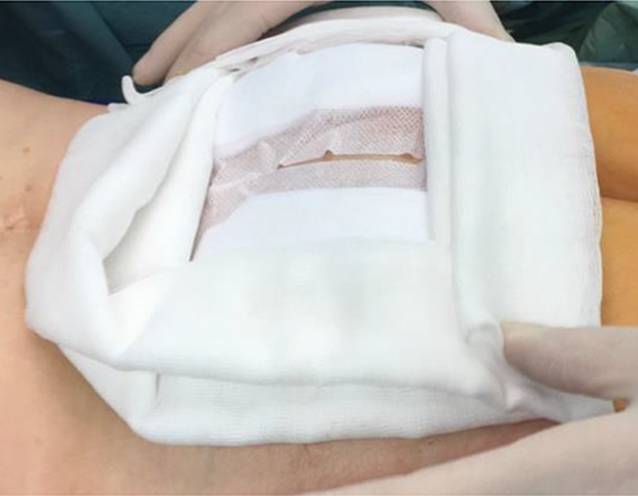

Nach der abschließenden Röntgenkontrolle erfolgt der Wundverschluss. Um den Behandlungserfolg sicherzustellen, ist ein adäquater Verschluss der Weichteile von essenzieller Bedeutung. Nach Überprüfung der Bluttrockenheit (Vermeidung postoperativer Hämatome) werden die gesicherte Faszie des M. glutaeus maximus und die thorakolumbale Faszie fest über der Platte verschlossen (11:15). Danach erfolgt der weitere schichtweise Wundverschluss. Ein Rahmenverband, der die Wundflächen vor Druckbelastungen schützen soll, wird abschließend angelegt (13:20) (Abb. 3). Die Patienten werden sofort postoperativ mit physiotherapeutischer Hilfe und entsprechender Analgetikatherapie mit schmerzadaptierter Vollbelastung mobilisiert.

## Postoperative Behandlung

**Figure 4:**
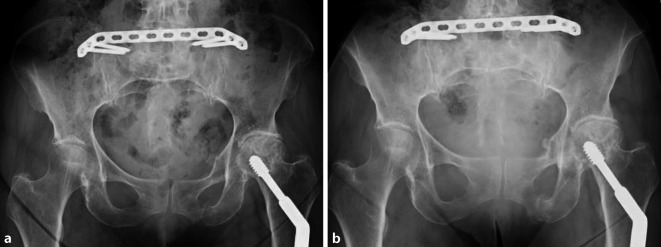

Sofortige Vollbelastung möglich. Anlage eines „Rahmenverbands“ zum Schutz der Wunde vor vermehrtem Druck. Des Weiteren werden die Patienten angehalten, längeres Liegen auf dem Rücken zu vermeiden. Die Entlassung kann üblicherweise ab dem 10. bis 12. postoperativen Tag und dann entweder in die Anschlussheilbehandlung oder nach Hause erfolgen. Nahtmaterialentfernung nach 12 bis 14 Tagen. Röntgenverlaufskontrolle vor Entlassung und nach 6 bis 8 Wochen. Wenn möglich, CT-Kontrolle nach 6 Monaten (Abb. 4).

## Fehler, Gefahren, Komplikationen

Essenziell sind ein weichteilschonendes Arbeiten bei der Präparation und ein Beachten der subfaszialen Plattenlage sowie der adäquate Faszienverschluss. Aus unserer Sicht ist es wesentlich, sich vor der Operation einen Überblick über die Mobilitätssituation der Patienten vor der Verletzung zu verschaffen. Für Patienten, die bereits vor der Verletzung nicht oder nur wenig mobil waren, eignet sich das Verfahren unserer Meinung nach nur bedingt, da Weichteilprobleme wahrscheinlicher sind.

Gefahren: intraforaminale Schraubenlage, Verletzung des Foramen sacrale (bei der Schaffung des Plattenlagers)

## Evidenz der Technik

Im Rahmen einer Nachuntersuchung wurden die Ergebnisse der Operationsmethode erfasst und insbesondere mit der iliosakralen Schraubenosteosynthese verglichen. Dabei zeigte sich eine vergleichbare Komplikationsrate bei deutlich niedriger Durchleuchtungszeit [11].

## Fazit für die Praxis

Die Versorgung der Insuffizienzfrakturen des Beckens durch eine minimal-invasive dorsale Beckenplatte ist bei korrekter Anwendung und Indikationsstellung eine gute Methode zur operativen Versorgung geriatrischer Patienten mit steiler Lernkurve und moderater Komplikationsrate.
